# Prognostic and therapeutic implications of disulfidptosis-related genes in multiple myeloma

**DOI:** 10.3389/fimmu.2025.1652179

**Published:** 2025-12-01

**Authors:** Yunke Zang, Peipei Zhou, Haotian Dong, Jingfei Wang, Rongxuan Cao, Guimao Yang, Qianqian Wu, Yanhua Sun, Yanli Sun

**Affiliations:** 1 School of Medical Laboratory, Shandong Second Medical University, Weifang, China; 2 Department of Hematology, Weifang People’s Hospital, Weifang, China; 3 Department of Laboratory Medicine, Affiliated Hospital of Shandong Second Medical University, Weifang, China

**Keywords:** disulfidptosis, multiple myeloma, prognostic signature, gene, risk stratification model

## Abstract

**Background:**

Multiple myeloma (MM), a malignancy of plasma cells in the bone marrow, urgently requires novel prognostic biomarkers. However, the prognostic significance of disulfidptosis-related genes and their association with treatment response in MM remain unclear.

**Methods:**

Transcriptomic data from MM samples were obtained from the Gene Expression Omnibus (GEO) database. A disulfidptosis-related prognostic model was constructed using LASSO-Cox regression analysis. The performance of the model was evaluated, and its clinical relevance to treatment response was subsequently assessed. Finally, the expression of the identified genes was validated by qRT-PCR and Western blotting.

**Results:**

Unsupervised cluster analysis identified a total of 121 differentially expressed genes. LASSO-Cox regression subsequently revealed a nine-gene prognostic signature comprising TPST2, HIF1A, KIF21B, MCPH1, MAST4, ANXA2, ALG14, PQLC3, and RANGAP1, which were used to establish and validate a robust risk stratification model. Partial validation demonstrated that ALG14, MCPH1, and PQLC3 were significantly downregulated, whereas TPST2 was markedly upregulated in MM cells.

**Conclusion:**

We established and validated a novel disulfidptosis-related prognostic model for MM, providing a potential biomarker for risk stratification and guidance for personalized therapeutic decisions.

## 1 Introduction

Disulfidptosis is a recently discovered form of regulated cell death driven by the abnormal accumulation of disulfide bonds. First identified in 2023 by Gan et al. and Chen et al. at the Anderson Cancer Center (1), this pathway is induced under glucose deprivation in cells with high expression of solute carrier family 7 member 11 (SLC7A11), a key cystine/glutamate antiporter (2). Under such conditions, depletion of the reducing agent nicotinamide adenine dinucleotide phosphate (NADPH) leads to excessive disulfide accumulation, which disrupts cytoskeletal integrity through abnormal disulfide cross-linking of actin filaments, ultimately resulting in cell death (3). Given its dependence on redox imbalance and cytoskeletal instability, disulfidptosis may play a critical role in malignancies characterized by oxidative stress and aberrant protein secretion.

Multiple myeloma (MM) is a prevalent hematologic malignancy characterized by the clonal expansion of plasma cells within the bone marrow. These malignant plasma cells secrete excessive monoclonal immunoglobulins and cytokines, resulting in osteolytic lesions, bone marrow suppression, and renal impairment (4). Although therapeutic advances, including proteasome inhibitors and immunomodulatory agents, have significantly improved patient outcomes, MM remains largely incurable due to inevitable relapse and the development of refractory disease (5). Therefore, identifying novel prognostic biomarkers and therapeutic targets is of critical importance. Notably, MM plasma cells produce abundant disulfide-rich immunoglobulins and misfolded proteins, suggesting a potential mechanistic link between disulfidptosis and MM pathogenesis. However, the prognostic significance of disulfidptosis-related genes (DRGs) in MM has yet to be elucidated.

In this study, we applied bioinformatics analyses and least absolute shrinkage and selection operator (LASSO)-Cox regression modeling to develop a DRG-based prognostic signature for MM. The predictive performance of the model was validated in an independent clinical cohort, while quantitative real-time polymerase chain reaction (qRT-PCR) and Western blot (WB) confirmed differential expression of DRGs in MM cells. Our findings provide some of the first evidence implicating disulfidptosis in MM biology, offering new insights into risk stratification and precision therapy.

## 2 Materials and methods

### 2.1 Data acquisition

**Figure 1:**
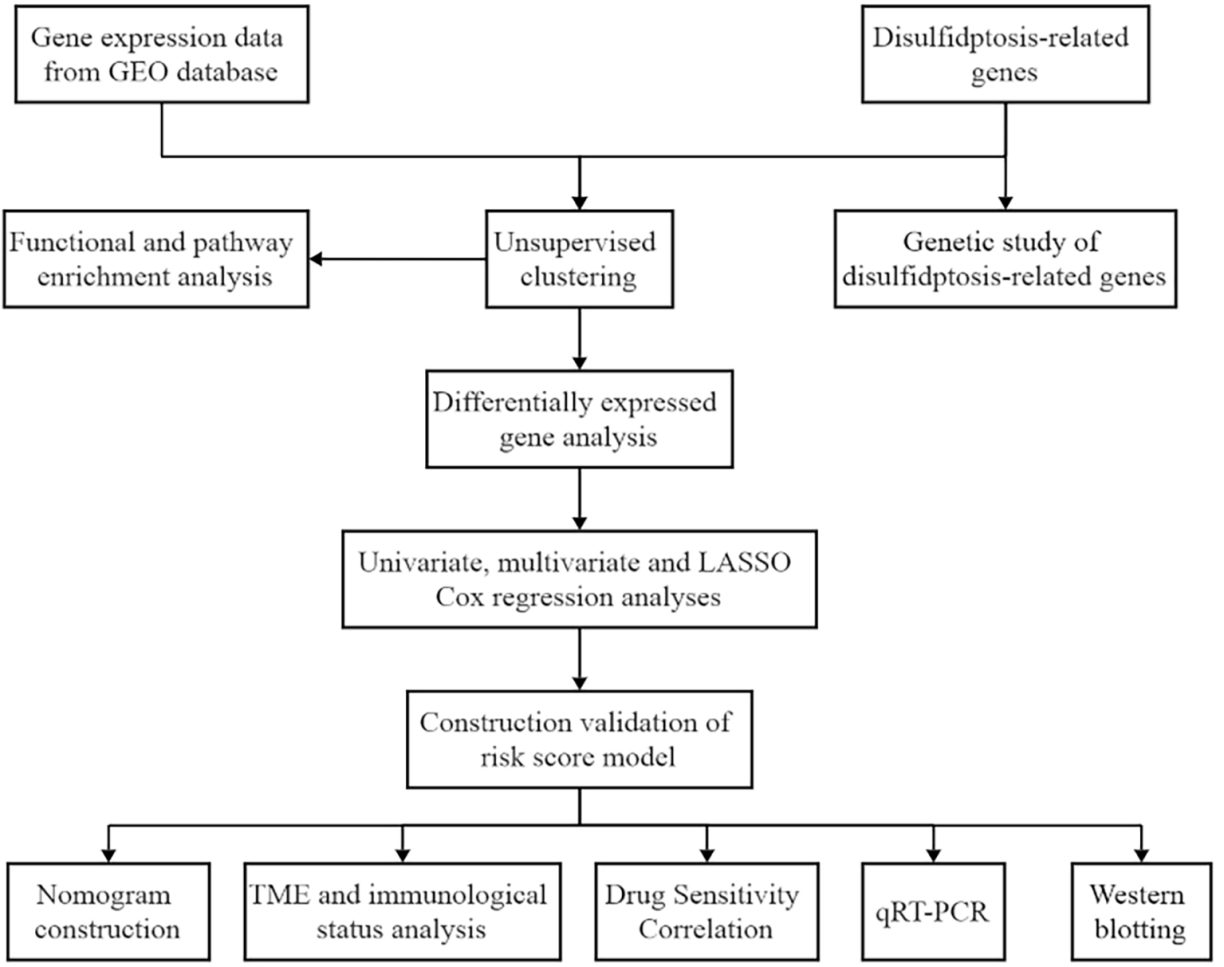
Flowchart of this study.

The overall study workflow is presented in **Figure 1**. Gene expression profiles of MM patients were retrieved from the Gene Expression Omnibus (GEO) database (https://www.ncbi.nlm.nih.gov/geo/) for the datasets GSE2658 and GSE57317, while normal sample data were obtained from GSE118985. All three datasets were generated using the Affymetrix Human Genome U133 Plus 2.0 Array (GPL570) platform. Raw microarray data were merged and batch-corrected using the “Combat” algorithm in the SVA package, yielding a unified dataset comprising 559 MM samples (GSE2658), 55 MM samples (GSE57317), and 68 normal samples (GSE118985) (see Additional file 1). To further assess the prognostic value of the model, dataset GSE136337 from GEO was used as an external validation cohort. DRGs were extracted based on the gene list reported by the Li research group (6).

### 2.2 Gene interaction network and genetic alterations

Interactions among DRGs were analyzed using the R packages “iGraph”, “Psych”, “Reshape2”, and “RColorBrewer”. Correlations among DRGs were examined with the “corrplot” package. Additionally, the “RCircos” package was used for localizing the position of genes on chromosomes. To explore protein-protein interactions (PPI), DRGs were submitted to the STRING database (version 12.0) (https://cn.string-db.org/), and the resulting network was analyzed (7). Somatic alteration frequencies and chromosomal mutation sites in MM were extracted from the cBioPortal for Cancer Genomics (http://www.cbioportal.org/). Furthermore, differential expression between MM and normal samples was assessed using the “limma” R package.

### 2.3 Unsupervised cluster analysis of differentially expressed genes

This study evaluated a total of 24 DRGs: ACTB, ACTN4, CAPZB, CD2AP, DSTN, FLNA, FLNB, GYS1, INF2, IQGAP1, LRPPRC, MYH10, MYH9, MYL6, NCKAP1, NDUFA11, NDUFS1, NUBPL, PDLIM1, RPN1, SLC3A2, SLC7A11, TLN1, and OXSM. The rationale for including each gene, encompassing its function, directionality, and relevance to disulfidptosis, is summarized in Additional File 2. Data were collected and analyzed using the “ConsensusClusterPlus” R package, which stratified the 614 tumor samples into two clusters (Cluster A and Cluster B). Survival differences between the clusters were assessed using the “survival” R package. Additionally, differences in clinical features between clusters were evaluated with the “pheatmap” R package.

### 2.4 Function and pathway enrichment analysis of DRGs

The “GSVA” and “GSEABase” R packages were used to investigate the underlying biological processes and signaling pathways. The datasets “c5.go.v2024.1.Hs.symbols.gmt” and “c2.cp.kegg_legacy.v2024.1.Hs.symbols.gmt” were obtained from the Molecular Signatures Database (MSigDB) (https://www.gsea-msigdb.org/gsea/msigdb/) as collections of annotated gene sets. A false discovery rate (FDR) < 0.05 was applied as the statistical threshold for clustering. Furthermore, single-sample gene set enrichment analysis (ssGSEA) was performed to quantify differences among the clustering classes, enabling a detailed comparison of their biological characteristics.

### 2.5 Construction and verification of disulfidptosis-related prognostic model

To quantitatively assess the relationship between MM and disulfidptosis, a disulfidptosis-related prognostic model was developed. The “limma” R package was employed to identify two distinct clusters, with filtering criteria set at FDR < 0.05 and absolute log_2_ fold change > 1. The complete set of MM samples (n = 614) was randomly divided into a training set (n = 307) and a validation set (n = 307) at a 1:1 ratio. DEGs between these subtypes were subjected to univariate Cox proportional hazards regression, with p < 0.05 considered statistically significant for prognostic relevance. LASSO-Cox regression was subsequently performed using the “glmnet” R package to construct a prognostic risk model associated with cell death (8). The optimal penalty parameter (λ) was determined via 10-fold cross-validation; λ.min, corresponding to the minimum cross-validation error, was selected for model construction, while λ.1se was also recorded as a reference for model robustness. Risk scores for disulfidptosis were calculated based on the expression profiles of key genes using the derived model equation. The training set was used to establish the prognostic model, and the validation set was employed to assess its predictive performance. The risk score was calculated according to the following formula:


$$\text{Risk score}=\sum_{\text{i}=1}^{\text{n}}=\text{ exp}\times \text{coef}$$

In the risk score formula, exp represents gene expression, and coef denotes gene risk coefficients. Samples were stratified into high-risk and low-risk groups based on the median risk score, following standard risk classification criteria. Kaplan-Meier (K-M) survival analysis was performed to evaluate the prognostic significance of the risk model, and receiver operating characteristic (ROC) curves were generated to assess its predictive accuracy. These analyses were conducted using the R packages “survival”, “survminer”, “pheatmap”, and “timeROC”. Principal component analysis (PCA) and t-distributed stochastic neighbor embedding (t-SNE) were also performed using the “Rtsne” and “ggplot2” packages to visualize differences between the two risk groups. Missing values were addressed via complete-case analysis, and potential outliers were examined using standardized residuals and leverage statistics, with exclusions applied only when justified. The proportional hazards assumption of the Cox models was tested using Schoenfeld residuals, and no significant violations were observed. Additionally, pathway enrichment scores for key biological processes were calculated using GSVA, and their correlations with the disulfidptosis risk score were assessed by Spearman correlation.

### 2.6 Construction and verification of the nomogram

A nomogram was constructed based on MM patient data to predict disease risk using the calculated risk scores. The results were visualized with the R packages “rms” and “rmda”. To further evaluate the clinical utility and robustness of the proposed model, its prognostic performance was compared with that of previously published MM risk models using the “survival” and “survcomp” packages. Decision curve analysis (DCA) was performed with the “rmda” package, and clinical impact curves (CICs) were generated to depict the number of patients classified as high risk and the corresponding true positives across different threshold probabilities.

### 2.7 Analysis of the tumor microenvironment and immune status

To analyze differences in the TME between the high-risk and low-risk groups associated with cell death, the “estimate” R package was used to calculate the immune score, stromal score, and estimate score. Gene set enrichment analysis (GSEA) was performed using the “clusterprofiler” R package to explore the underlying biological pathways and mechanisms. The CIBERSORT algorithm (https://cibersortx.stanford.edu/) was implemented in R to evaluate 22 immune cell types per TME sample (9), alongside an analysis of their relationships in immune cell infiltration. MCP-counter and xCell were additionally employed to provide orthogonal assessments of immune and stromal cell populations, thereby enhancing robustness and capturing bone marrow-specific features. Spearman correlation analysis was conducted to assess the relationship between the risk model gene signatures and immune infiltration levels. To investigate potential factors contributing to tumor immune evasion, the tumor immune dysfunction and exclusion (TIDE) algorithm (http://tide.dfci.harvard.edu/) was applied. A heatmap was generated to visualize correlations between immune checkpoint genes and risk scores. Immune checkpoint genes significantly associated with risk scores (p < 0.05) were further subjected to FDR correction and multivariable Cox regression to evaluate their independent prognostic significance, with hazard ratios and 95% confidence intervals (CIs) reported and visualized in forest plots. K-M survival analysis was also performed using the “survival” and “survminer” R packages to assess their prognostic relevance.

### 2.8 Analysis of drug sensitivity

Expression and sensitivity data for targeted drugs were obtained from the Genomics of Drug Sensitivity in Cancer (GDSC) database (https://www.cancerrxgene.org/). To identify statistically significant differences in drug response, a threshold of P < 0.001 was applied. The “oncopredict” and “parallel” R packages were used to assess differences in drug sensitivity between the two risk groups (10). This analysis allowed prediction of individualized treatment responses and highlighted potential therapeutic agents for risk-stratified MM patients.

### 2.9 Verification of gene expression in normal cells and tumor cells

The MM cell lines RPMI8226 and U266 were obtained from Baidi Biotechnology Co., Ltd. (Zhejiang, China). Cells were cultured in RPMI 1640 medium (GIBCO, USA) supplemented with 20% fetal bovine serum (FBS) and 1% penicillin/streptomycin at 37°C in a 5% CO_2_ atmosphere. Bone marrow aspirates were collected from 13 MM patients and 4 healthy controls at Weifang People’s Hospital. Normal plasma cells and patient-derived myeloma plasma cells were isolated from bone marrow mononuclear cells using CD138^+^ magnetic bead selection. The clinical characteristics of the MM patients are summarized in Additional File 3. This study was approved by the Ethics Committee of Shandong Second Medical University, and informed consent was obtained from all participants. Total RNA was extracted using TRIzol reagent (Sparkjade, China) following the manufacturer’s protocol. Gene expression was quantified by qRT-PCR using the SYBR Green method on an Applied Biosystems 7500 Fast Real-Time PCR system. Relative expression levels were calculated using the 2^-△△Ct^ method, with GAPDH serving as the housekeeping gene for normalization. The primers used for qRT-PCR are listed in **Table 1**.

**Table 1:** Primers used for qRT‐PCR.

Gene name	Strand	5’-3’
MCPH1	Forward	ATGTAGTGGCCTATGTTGAAGTG
	Reverse	CCACAAGCTGTGTTGTAAATGTC
PQLC3	Forward	CCTCATCGCGCAAGATGTCAT
	Reverse	CCAAGAAGACACCAATACAGCG
TPST2	Forward	CCCCTTCGTCATCAACAACAC
	Reverse	GAGCTTCCTAAGTGGGAGGAG
ALG14	Forward	CCTGCGAATATGGGTAGTGCT
	Reverse	CTCAGTGGTATGCCCACCG
RANGAP1	Forward	CAGGCTTTCGCTGTCAACC
	Reverse	GCAGCATCCCTCTTGATTTCAC
KIF21B	Forward	ACCTATGACTTTGTCTTCGACCT
	Reverse	CAGCACCGTGGCATTATAGC

WB analysis was subsequently performed. Equal amounts of cell lysates were loaded onto SDS-PAGE gels for electrophoresis, and proteins were then transferred onto polyvinylidene fluoride (PVDF) membranes. Membranes were blocked with 5% skim milk in Tris-buffered saline containing 0.1% Tween-20 (TBST) and incubated overnight with primary antibodies. Following this, membranes were incubated with HRP-conjugated goat anti-rabbit secondary antibody for 1 h. Specific protein bands were visualized using an enhanced chemiluminescence reagent and an Amersham Imager 600 (GE Healthcare Biosciences, Pittsburgh, PA, USA). Band intensities were quantified using ImageJ software (v.1.50a) (https://imagej.net/software/imagej/).

### 2.10 Statistical analyses

All data analyses and visualizations were performed via R (version 4.4.1) and GraphPad Prism 9.5. For quantitative variables, Student’s t test was used to evaluate differences between groups with normally distributed data, whereas the Wilcoxon test was used for skewed data, with a P value of less than 0.05 considered statistically significant. In this context, the levels of significance are represented as follows: * *P* < 0.05, ** *P* < 0.01, *** *P* < 0.001, and **** *P* < 0.0001. All genome-wide and multi-gene P values were adjusted by the FDR method, with FDR < 0.05 considered significant.

## 3 Results

### 3.1 Genetic characterization of DRGs in MM

**Figure 2:**
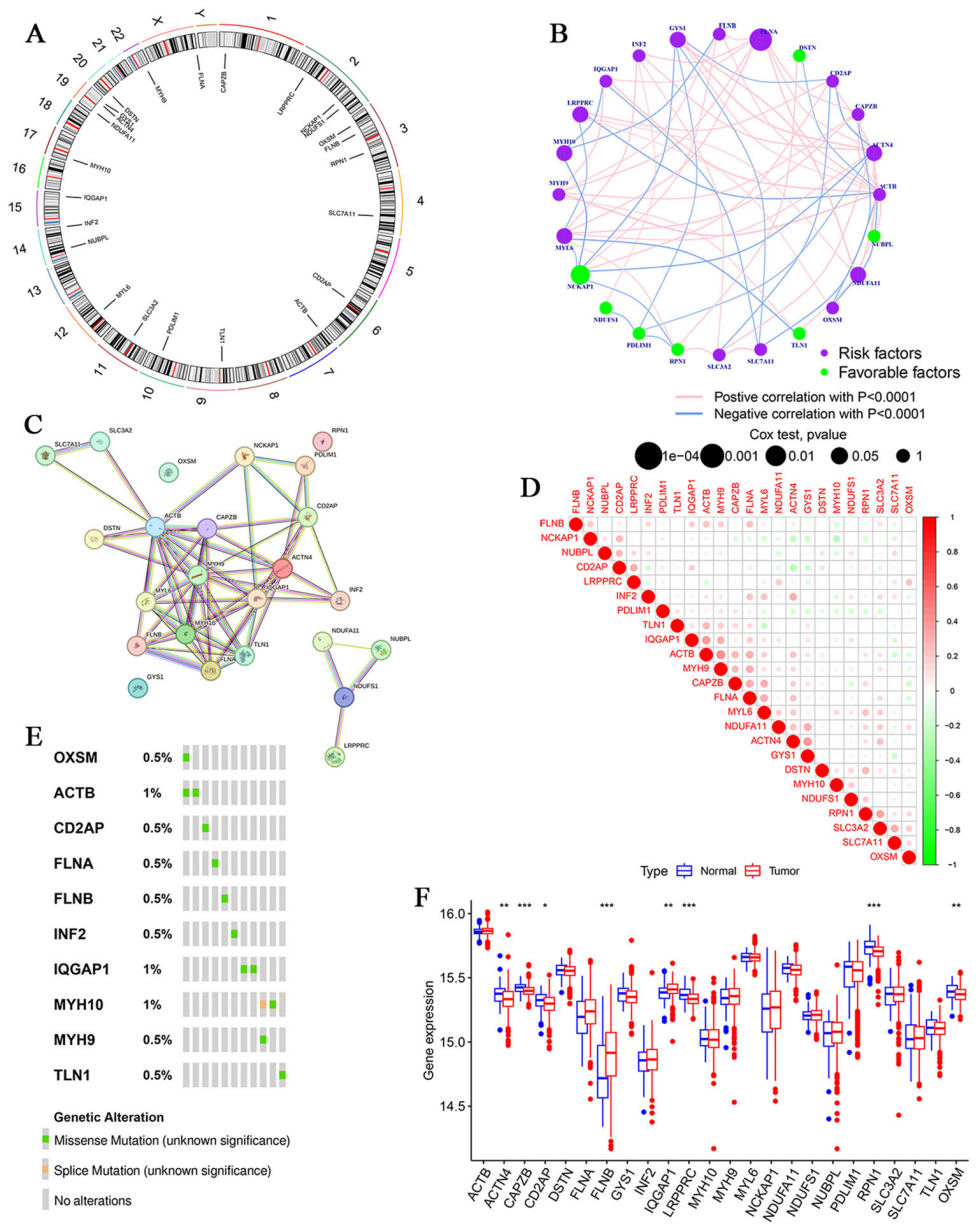
Genetic alterations of DRGs in MM. **(A)** Distribution of DRGs on chromosomes. **(B)** Comprehensive network map combining disulfidptosis-regulatory gene interactions and prognosis. **(C)** The PPI network downloaded from the STRING database indicates the interactions among the 24 candidate genes. **(D)** Spearman correlations between genes. The color green represents negative regulation; the color red represents positive regulation. **(E)** Genetic changes in DRGs in MM. **(F)** Expression of 24 DRGs in MM tissue versus normal tissue. (*P<0.05,**P<0.01, and ***P<0.001).

Chromosomal mapping of the 24 DRGs revealed their genomic distribution (**Figure 2A**). Regulatory network analysis demonstrated their interactions, suggesting potential prognostic relevance in MM (**Figure 2B**). Correlation analysis identified strong positive associations (e.g., ACTB-MYH9, r = 0.41) and negative associations (e.g., ACTN4-CD2AP, r = -0.26) among DRGs (**Figures 2C, D**). Genomic profiling detected missense and splice-site mutations in MM samples (**Figure 2E**). Differential expression analysis of GEO datasets revealed significant downregulation of ACTN4, CAPZB, CD2AP, DSTN, GYS1, LRPPRC, MYL6, PDLIM1, RPN1, and SLC3A2 in MM tissues compared with controls, whereas ACTB, FLNA, FLNB, INF2, IQGAP1, MYH9, NCKAP1, NUBPL, and SLC7A11 were upregulated (**Figure 2F**), implicating DRGs in MM pathogenesis.

### 3.2 Disulfidptosis-based molecular subtyping of MM

**Figure 3:**
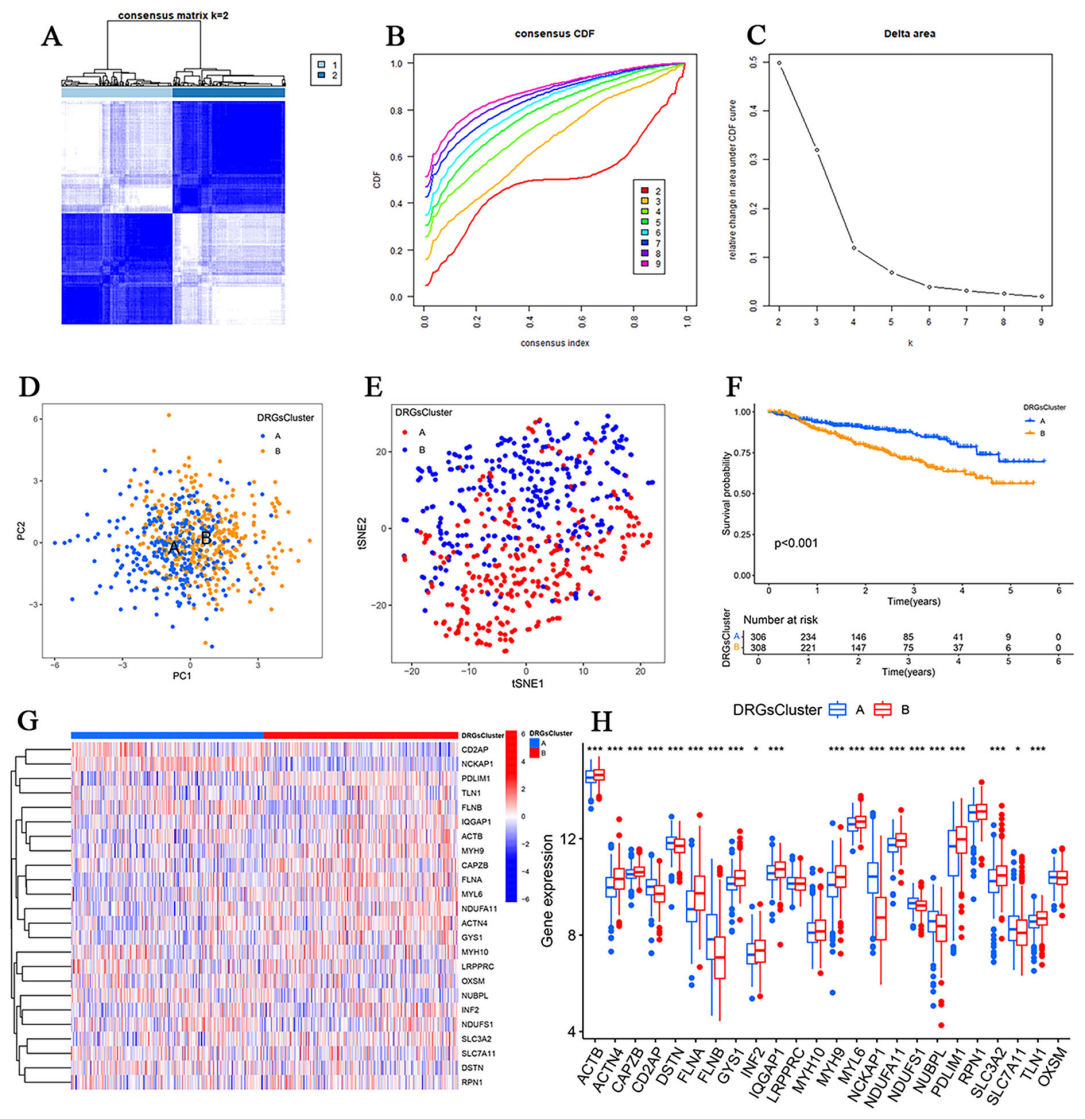
Consensus clustering of DRGs in MM. **(A–C)** Consensus matrix heatmap of cohort samples defining two clusters (k = 2). **(D, E)** PCA and t-SNE of the consensus matrix when k = 2. F Kaplan-Meier survival curves revealed significant differences between the two subtypes (P < 0.001). **(G, H)** Expression of DRGs in the two clusters. (*P<0.05,**P<0.01, and ***P<0.001).

Consensus clustering of 614 MM samples (GSE2658 and GSE57317) identified two robust subtypes (k = 2), which were validated by cumulative distribution function (CDF) curves (**Figures 3A–C**). Cluster A (n = 306) and Cluster B (n = 308) exhibited clear separation in principal component analysis (PCA) and t-distributed stochastic neighbor embedding (t-SNE) plots (**Figures 3D, E**). Survival analysis revealed significantly longer overall survival (OS) in Cluster A compared with Cluster B (P < 0.001; **Figure 3F**). Cluster A was characterized by upregulation of CD2AP, DSTN, FLNB, NCKAP1, NDUFS1, and NUBPL, whereas Cluster B overexpressed MYL6, NDUFA11, PDLIM1, SLC3A2, and TLN1 (**Figures 3G, H**).

### 3.3 Functional enrichment of DRG-associated pathways

**Figure 4:**
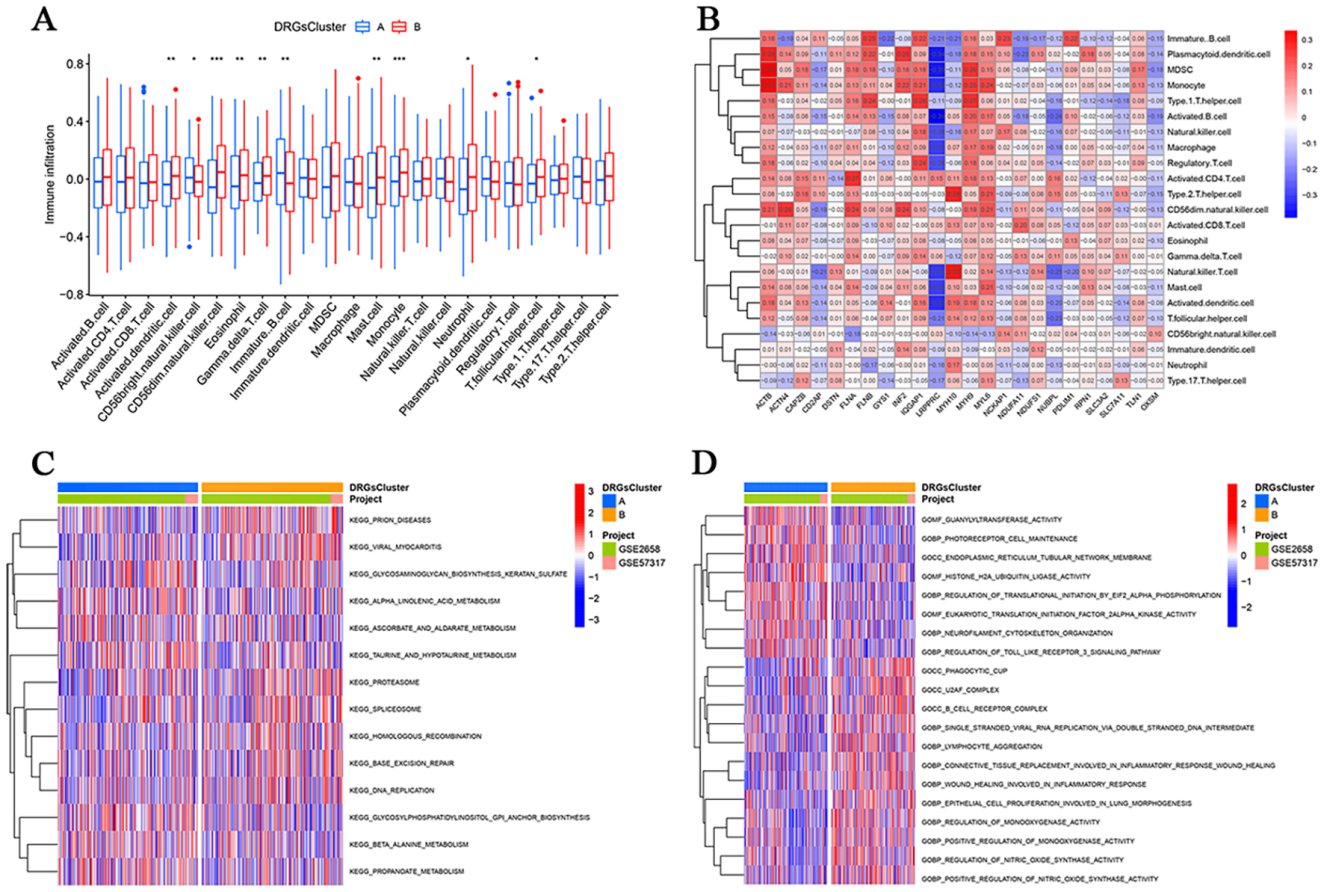
Biological properties of two subtypes of MM. **(A)** Differences in immune infiltration between the disulfidptosis subgroups according to the ssGSEA algorithm. **(B)** Differences in the infiltration of 23 immune cells between MM subtypes. **(C)** Biological pathway analysis between the two disulfidptosis clusters. **(D)** Multiple pathway analysis between the two disulfidptosis clusters. (*P<0.05,**P<0.01, and ***P<0.001).

ssGSEA indicated higher immune infiltration in Cluster B, with CD56dim NK cells and monocytes significantly enriched (P < 0.001; **Figure 4A**). Immune cell correlation networks highlighted interactions with cell death-related genes (**Figure 4B**). Gene set variation analysis (GSVA) revealed that Cluster B was enriched in pathways related to prion diseases, viral myocarditis, DNA repair, and replication, whereas Cluster A exhibited enrichment in guanylyltransferase activity and histone ubiquitination pathways (**Figures 4C, D**).

### 3.4 Development and validation of a disulfidptosis prognostic model

**Figure 5:**
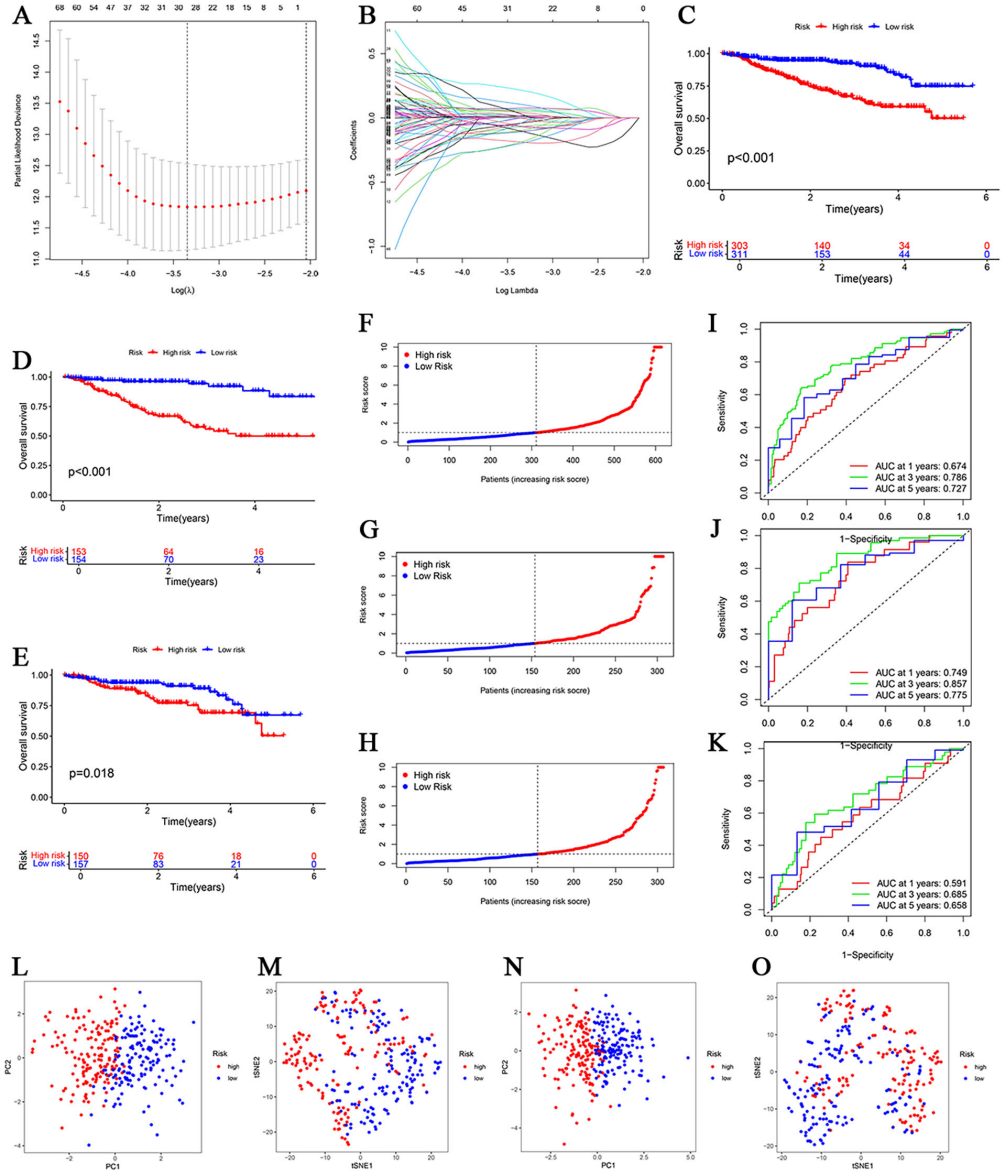
Establishment and verification of the disulfidptosis-related signature. **(A)** Construction of the prognostic model via Least absolute shrinkage and selection operator (LASSO). **(B)** LASSO coefficient profiles of candidate DEGs. **(C–E)** Survival curves for the risk score groups in the total sample, training, and validation data. **(F–H)** ROC curves for the total sample, training, and validation data. **(I–K)** Survival status distribution for risk score groups in the total sample, training, and validation data. **(L, M)** PCA and t-SNE analyses of the samples in training data. **(N, O)** PCA and t-SNE analyses of the samples in the validation data.

From 690 initial DEGs, univariate Cox regression identified 121 prognostic genes. DEGs were determined exclusively within the training set, and a Lasso-Cox model with 10-fold cross-validation was applied to select the optimal penalty parameter (λ.min ≈ 0.051), narrowing the candidates to 29 genes. Subsequent multivariate regression yielded a 9-gene prognostic signature: Risk score = (0.4500 × TPST2) + (0.1514 × HIF1A) + (0.1571 × KIF21B) + (-0.6397 × MCPH1) + (-0.5123 × MAST4) + (0.3354 × ANXA2) + (-0.4341 × ALG14) + (-0.2931 × PQLC3) + (0.4405 × RANGAP1). The results of the multivariate regression analysis are shown in **Supplementary Figure 1** and **Additional File 4**. High-risk patients exhibited significantly worse OS in the combined cohort (P < 0.001), training set (P < 0.001), and validation set (P < 0.05; **Figures 5C–H**). ROC analysis confirmed the prognostic accuracy of the model, with areas under the curve (AUCs) of 0.674, 0.786, and 0.727 at 1, 3, and 5 years, respectively (**Figures 5I–K**). Validation in the external dataset GSE136337 yielded AUCs of 0.646, 0.705, and 0.735 at 1, 3, and 5 years, respectively (**Supplementary Figure 2**), demonstrating high predictive performance. Furthermore, PCA and t-SNE analyses revealed clear separation between the high- and low-risk groups, confirming distinct risk stratification (**Figures 5L–O**).

### 3.5 Association of the disulfidptosis risk score with core biological pathways

To elucidate the biological implications of the disulfidptosis-related signature, we examined its correlation with key pathways involved in MM pathogenesis and disulfidptosis mechanisms. Spearman correlation analysis was performed between the risk score and pathway enrichment scores representing proteasome activity, endoplasmic reticulum (ER) stress, oxidative phosphorylation, and actin cytoskeleton organization. Notably, the risk score was significantly positively correlated with proteasome activity (r = 0.34, P < 0.001) and oxidative phosphorylation (r = 0.28, P < 0.001) (**Supplementary Figure 3**). Conversely, a significant negative correlation was observed with the ER stress response (r = -0.16, P < 0.001), and a weak but statistically significant inverse correlation was found with actin filament organization (r = -0.09, P < 0.05). These findings suggest that high-risk patients, as defined by our model, exhibit enhanced proteasomal function and metabolic activity, accompanied by a dampened ER stress response and subtle alterations in actin cytoskeletal dynamics.

### 3.6 Construction and validation of the disulfidptosis-based nomogram

**Figure 6:**
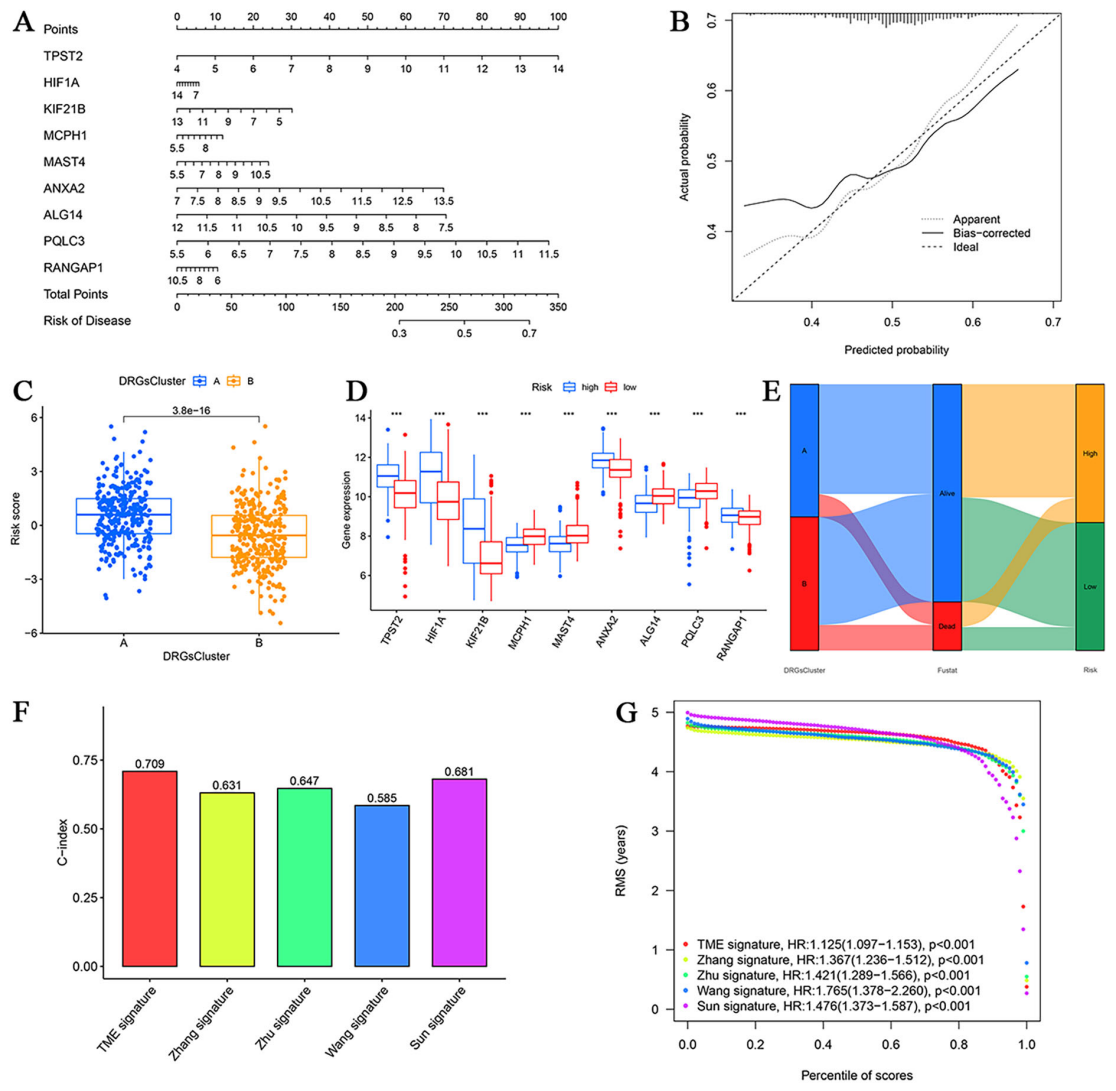
Nomogram construction. **(A)** MM risk prediction nomogram. **(B)** Calibration curve of the nomogram. **(C)** Differences in risk scores between the two clusters. **(D)** Differential expression of disulfidptosis regulators in the two risk groups. **(E)** Association between disulfidptosis subtypes, survival status, and disulfidptosis risk groups. **(F)** Comparison of the C-index values of the disulfidptosis trait model with those of the four MM trait models. **(G)** RMS curves of the disulfidptosis characteristic model versus the four MM characteristic models. (***P<0.001).

To improve prognostic precision in MM patients, we constructed a nomogram incorporating the disulfidptosis-related risk score (**Figure 6A**). Bootstrap validation with 1,000 resamples demonstrated excellent concordance between predicted and observed outcomes, as indicated by the calibration curve (average absolute error = 0.022), confirming the model’s robustness (**Figure 6B**). Comparative analysis revealed significantly lower risk scores in Cluster B compared with Cluster A (P < 0.001), highlighting the model’s discriminative ability (**Figure 6C**). Differential gene expression patterns between the high- and low-risk groups further validated the biological relevance of the cell death-related model (**Figure 6D**). Decision curve analysis (DCA) indicated that the nomogram provided superior net benefit across a range of threshold probabilities, and clinical impact curves (CICs) confirmed that predicted high-risk cases closely matched actual outcomes, supporting its potential clinical utility (**Supplementary Figure 4**).

The Sankey diagram illustrated consistency in patient stratification across classification methods, with Cluster A predominantly comprising high-risk patients and Cluster B enriched for low-risk cases (**Figure 6E**). In benchmark comparisons with four established MM prognostic models (11–14), our disulfidptosis-based model demonstrated superior performance, exhibiting significant survival discrimination across all comparative models (P < 0.05; **Supplementary Figures 5A–D**) and higher predictive accuracy (C-index: 0.709 *vs*. Sun: 0.681, Zhu: 0.647, Zhang: 0.631, Wang: 0.585; P < 0.05; **Figures 6F, G**; **Supplementary Figures 5E–G**). Collectively, these results establish the disulfidptosis signature as a clinically actionable prognostic tool with enhanced stratification power relative to existing models.

### 3.7 Characterization of TME, immune infiltration, and immune checkpoints across risk groups

**Figure 7:**
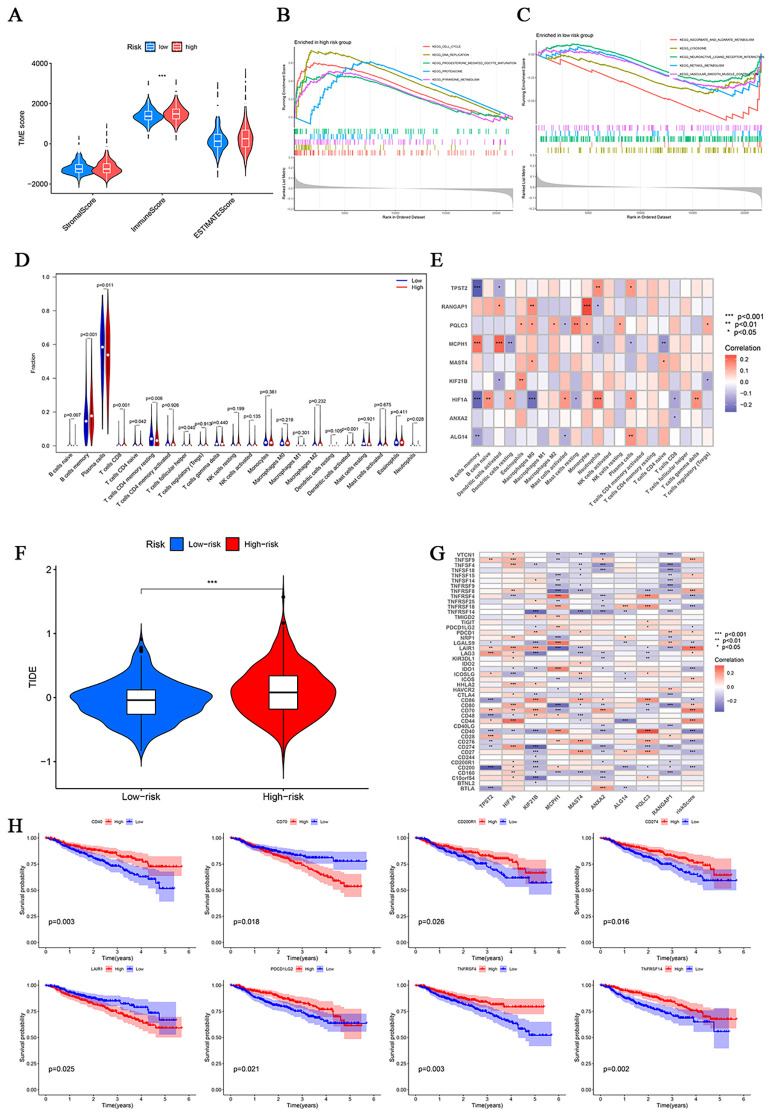
TME cell infiltration and tumor immune in ltration analysis with disulfidptosis risk score. **(A)** Differences in the stromal score, immune score and ESTIMATE score between subtypes. **(B, C)** GSEA of genes with high and low expression. **(D)** Infiltration abundance of 22 tumor immune cells in the high- and low-risk groups. **(E)** Heatmap of correlations between genes and infiltrating immune cells. **(F)** TIDE scores in different risk groups. **(G)** Correlation analyses of immune checkpoints. **(H)** Survival curves for immune checkpoint-related genes. (*P<0.05,**P<0.01, and ***P<0.001).

We applied the Estimation of Stromal and Immune cells in Malignant Tumor Tissues Using Expression Data (ESTIMATE) algorithm to assess TME characteristics, including stromal, immune, and combined estimate scores. Comparative analysis revealed significantly higher immune scores in high-risk patients (P < 0.001), suggesting increased immune cell infiltration (**Figure 7A**).

To further investigate functional differences between high- and low-risk groups, GSEA was performed to identify pathways associated with risk stratification. High-risk samples were significantly enriched in pathways related to the cell cycle, DNA replication, progesterone-mediated oocyte maturation, proteasome activity, and pyrimidine metabolism. In contrast, low-risk samples exhibited enrichment in pathways associated with ascorbate and aldarate metabolism, lysosome function, neuroactive ligand-receptor interaction, retinol metabolism, and vascular smooth muscle contraction (P < 0.05; **Figures 7B, C**).

To characterize the immune landscape of the TME, we applied the CIBERSORT algorithm to deconvolute 22 immune cell subsets across all samples. This analysis consistently revealed enrichment of memory B cells and plasma cells throughout the cohort, with high-risk patients exhibiting significantly higher infiltration of memory B cells, CD8^+^ T cells, and activated dendritic cells compared with low-risk patients (P < 0.001; **Figure 7D**; **Supplementary Figure 6**). Correlation analysis identified the strongest negative associations between risk scores and both memory B cells and activated dendritic cells, whereas naive B cells and plasma cells showed the most pronounced positive associations; Spearman correlation further confirmed a significant relationship between the gene signature and infiltrating immune cells (**Figure 7E**). Sensitivity analyses using MCP-counter and xCell revealed consistent patterns: high-risk samples exhibited increased tumor/plasma-cell signals alongside reduced B-lineage cells, CD8^+^ T cells, cytotoxic lymphocytes, and NK cells. MCP-counter additionally highlighted alterations in neutrophils, monocytic lineages, endothelial cells, and fibroblasts. Notably, plasma-cell signatures displayed an inverse relationship with normal B-cell signals, consistent with the expectation that malignant plasma-cell expansion suppresses normal B-cell states (**Supplementary Figure 7**). Collectively, these findings show that the tumor microenvironment in high-risk patients is characterized by an abundance of malignant plasma cells and a scarcity of key effector immune cells, thereby fostering an immunosuppressive milieu associated with a poor prognosis.

TIDE analysis revealed significantly higher scores in high-risk patients (P < 0.001), suggesting increased potential for immune evasion and reduced responsiveness to immunotherapy (**Figure 7F**). Hotspot analysis further demonstrated correlations between the disulfidptosis-related gene signature and immune checkpoint genes (**Figure 7G**). K-M survival analysis indicated that higher expression of checkpoint genes such as CD70 and LAIR1 was associated with improved survival within the high-risk group, whereas elevated expression of CD40, CD200R1, CD274, PDCD1LG2, TNFRSF4, and TNFRSF14 was correlated with significantly better outcomes in intermediate- and high-risk patients compared with low-risk individuals (P < 0.05; **Figure 7H**). Multivariable Cox regression analysis of all immune checkpoint genes is shown in **Supplementary Figure 8**. Notably, CD274 exhibited a hazard ratio (HR) of 0.73 (95% CI, 0.50-1.06), indicating a non-significant trend toward reduced mortality with higher expression, consistent with the K-M results but not reaching statistical significance. This observation may reflect biological heterogeneity, as CD274 can mark an inflamed, T-cell-rich microenvironment associated with better prognosis, measurement variability, or residual confounding factors such as tumor burden or prior treatment, and thus should be interpreted cautiously.

### 3.8 Drug sensitivity analysis based on disulfidptosis risk stratification

**Figure 8:**
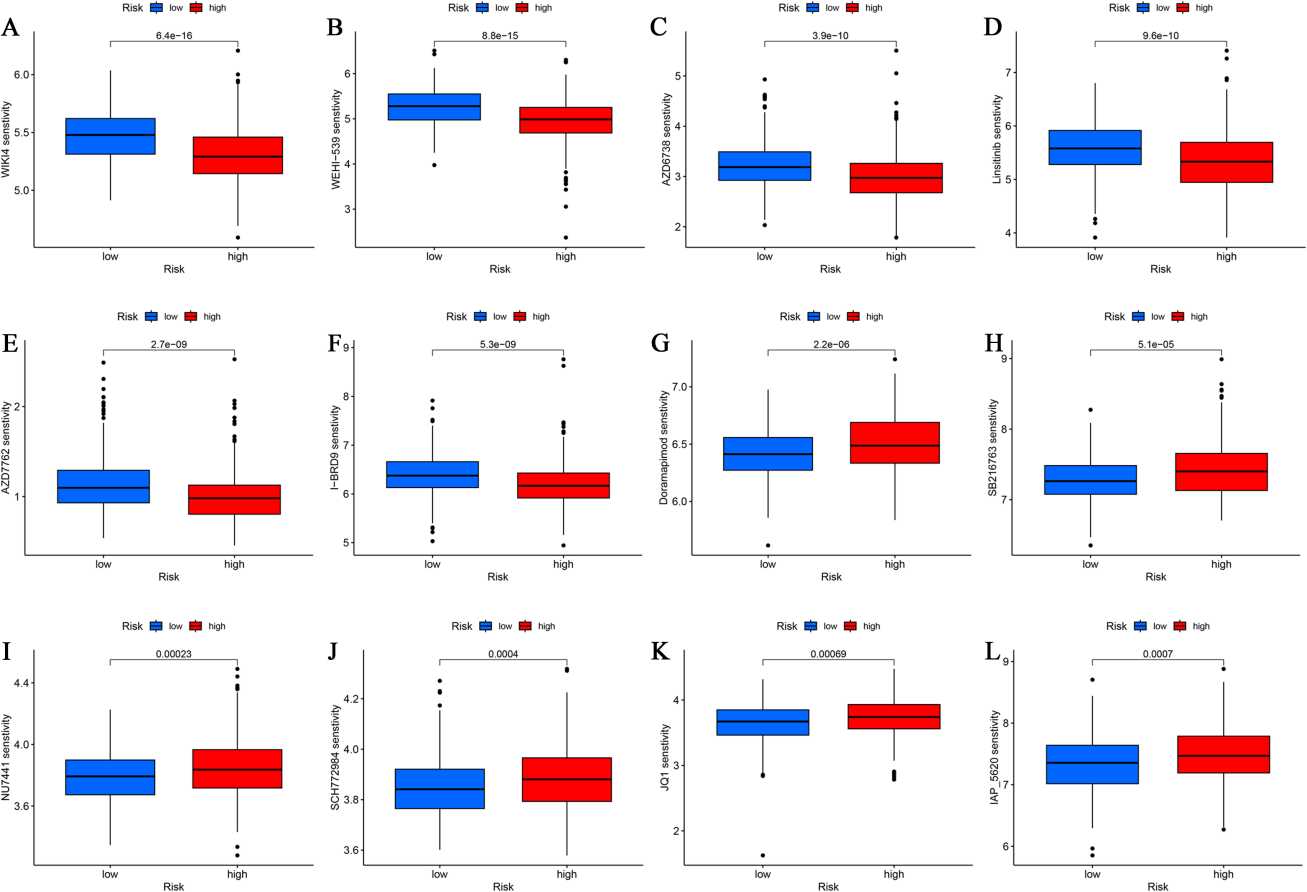
Relationships between risk groups and drug sensitivity. **(A–L)** Estimated IC50 values of common drugs in patients with high and low disulfidptosis scores.

To evaluate the clinical applicability of our risk model, we assessed correlations between disulfidptosis-related risk scores and therapeutic responses using the oncoPredict algorithm for 198 FDA-approved and experimental antitumor agents. To ensure reliable drug sensitivity predictions, expression data were subjected to strict preprocessing and quality control. PCA revealed no obvious batch effects, and expression distributions were highly consistent across samples (**Supplementary Figure 9**), confirming dataset comparability and suitability for downstream oncoPredict modeling. Significant differences in predicted drug sensitivity were observed between risk groups (P < 0.05). Specifically, low-risk patients exhibited increased sensitivity to WIKI4, WEHI-539, AZD6738, Linsitinib, AZD7762, and I-BRD9, whereas high-risk patients were more responsive to Doramapimod (BIRB 796), SB216763, NU7441, SCH772984, JQ1, and IAP_5620 (**Figure 8**). These findings highlight the potential of the risk model for precision oncology applications, with risk stratification predicting distinct therapeutic vulnerabilities. The observed differential sensitivity profiles suggest mechanistic differences in drug response pathways between disulfidptosis-defined subgroups.

### 3.9 Experimental validation of the prognostic signature

**Figure 9:**
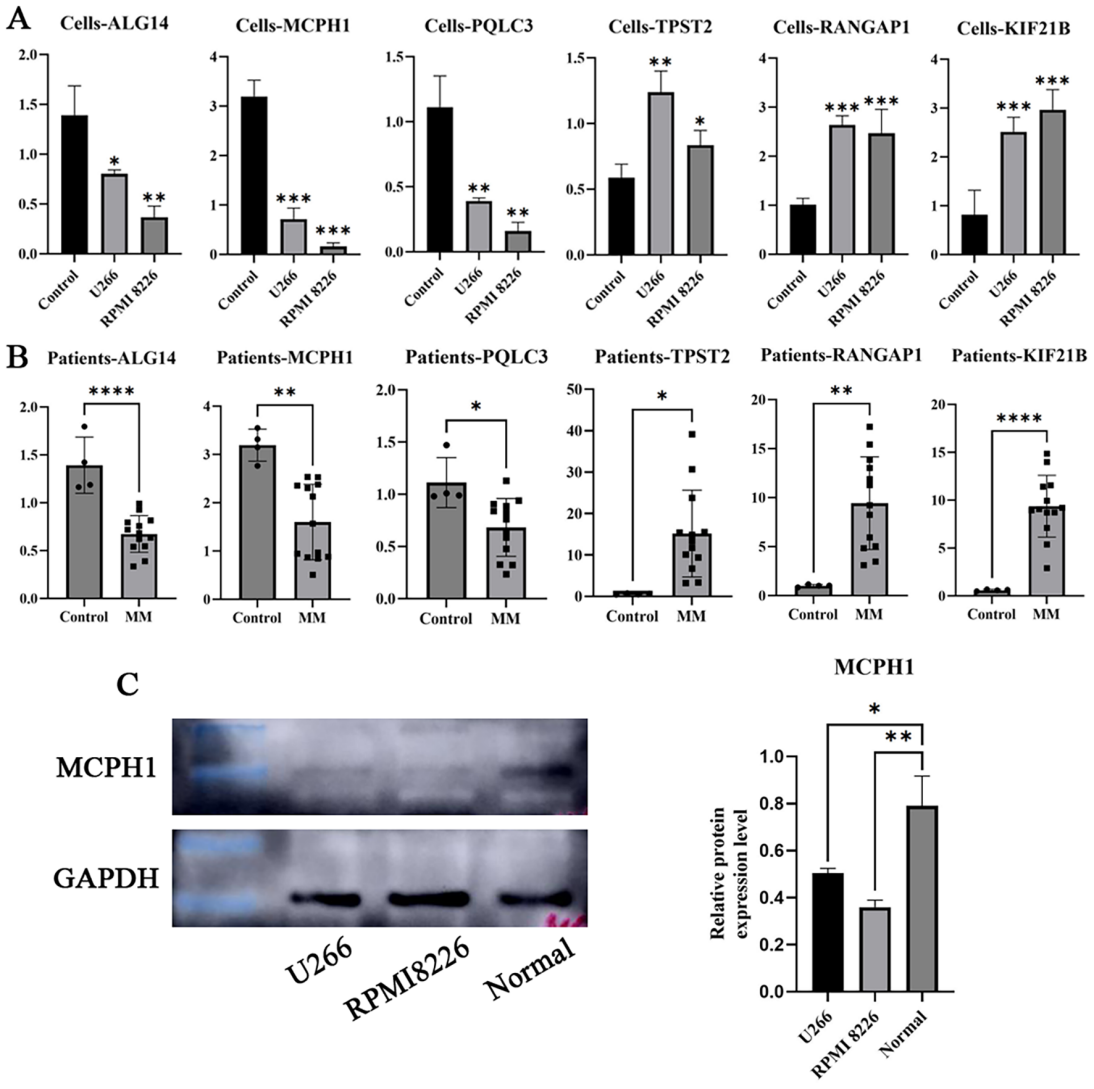
External experimental validation of the prognostic signature. **(A)** qRT-PCR was used to validate the DRGs expression in MM cell lines (RPMI8226 and U266) (Mean ± SEM). **(B)** qRT-PCR validated the DRGs expression in MM patients and control samples (Mean ± SEM). **(C)** WB analysis validated the protein expression levels of MCPH1 (The blots are cropped from different parts of the same gel, and the original blots are presented in **Supplementary Figure 10**). (*P<0.05, **P<0.01, ***P<0.001, and ****P<0.0001).

To validate the clinical relevance of our prognostic signature, we performed systematic molecular characterization of the nine identified key genes. Previous studies have reported upregulation of ANXA2 and HIF1A in MM, whereas MAST4 exhibits tumor-suppressive downregulation. We therefore focused experimental validation on the remaining six genes (MCPH1, PQLC3, TPST2, ALG14, RANGAP1, and KIF21B), whose roles in MM pathogenesis remain uncharacterized. qRT-PCR analysis of MM cell lines (RPMI8226 and U266) compared with normal plasma cells revealed significant downregulation of ALG14, MCPH1, and PQLC3, and marked upregulation of TPST2, RANGAP1, and KIF21B (**Figure 9A**). These expression changes were confirmed in paired primary MM samples (n = 13) by paired t-test, with all comparisons reaching statistical significance (P < 0.05; **Figure 9B**). Considering MCPH1’s established tumor-suppressor function in other malignancies, we performed WB analysis, which demonstrated reduced MCPH1 expression in MM cell lines relative to controls (**Figure 9C**; uncropped images are shown in **Supplementary Figure 10**), suggesting that loss of MCPH1 may contribute to MM pathogenesis. This multi-level validation confirms the biological relevance of our prognostic signature and identifies MCPH1 as a potential novel tumor suppressor in MM.

## 4 Discussion

Multiple myeloma is characterized by the clonal expansion of malignant plasma cells within the bone marrow, resulting in their pathological accumulation. Despite substantial therapeutic advancements over the past decade, including proteasome inhibitors, immunomodulatory agents, and monoclonal antibodies, the prognosis for MM patients remains poor, highlighting the need for improved prognostic and therapeutic assessment strategies (15). Several regulated cell death pathways, including apoptosis (16), autophagy (17), ferroptosis (18), and cuproptosis (19), have been implicated in MM pathogenesis. Recently, disulfidptosis has emerged as a novel form of programmed cell death driven by intracellular disulfide accumulation, which disrupts the F-actin cytoskeleton and induces cytoskeletal collapse. Emerging evidence suggests that disulfidptosis contributes to tumorigenesis and treatment response across multiple cancers, including lung (20), breast (21), and bladder (22) cancers, as well as hematologic malignancies such as acute myeloid leukemia (23) and diffuse large B-cell lymphoma (24). However, the specific relationship between disulfidptosis and MM prognosis or drug resistance remains largely unexplored and warrants further investigation.

To address this gap, we derived a nine-gene disulfidptosis-related signature (TPST2, HIF1A, KIF21B, MCPH1, MAST4, ALG14, PQLC3, RANGAP1, and ANXA2) and evaluated its clinical and biological associations. Compared with established MM classifiers such as UAMS/GEP70 and SKY92 (25, 26), which primarily capture proliferation, chromosomal instability, and plasma-cell differentiation programs, our signature emphasizes disulfide stress-related biology and cytoskeletal vulnerability, potentially providing complementary prognostic information. Comparison with published signatures for cell death and post-translational modifications showed minimal overlap with our nine DRGs at the gene level (13, 27–32). In summary, our signature captures mechanisms largely independent of known classifiers and thus points to novel biology that may inform MM prognosis and immune regulation.

The mechanism of disulfidptosis involves SLC7A11-mediated cystine uptake, NADPH depletion, and aberrant disulfide cross-linking in actin, culminating in F-actin contraction and cytoskeletal collapse (33). In contrast to ferroptosis, which features GPX4-regulated iron-dependent lipid peroxidation, and ER stress/UPR-mediated death, associated with proteostasis burdens, the features of disulfidptosis represent a fundamentally distinct form of cell death³³. SLC7A11 can modulate multiple redox-sensitive pathways, which accounts for the observed partial overlap. Nevertheless, the DRG signature identifies mechanistic dimensions not fully captured by existing ferroptosis or UPR models, thereby highlighting potential complementary therapeutic vulnerabilities.

Building on these mechanistic and comparative insights, we developed a prognostic risk model based on nine DRGs identified through univariate, multivariate, and LASSO-Cox regression analyses. The model demonstrated strong predictive performance, with AUC values of 0.674, 0.786, and 0.727 for 1-, 3-, and 5-year survival, respectively, reflecting robust discriminative ability. Furthermore, a nomogram incorporating these genes exhibited high accuracy for MM prognosis, supporting its potential utility as a clinical decision-making tool.

The significant correlations observed between the disulfidptosis-derived risk score and key biological pathways provide strong support for its mechanistic relevance in multiple myeloma. The pronounced positive correlation with proteasome activity highlights the model’s ability to identify tumors exhibiting heightened dependence on protein degradation, a recognized feature of aggressive disease and a cornerstone of current therapy. Conversely, the inverse correlation with the ER stress response suggests that high-risk tumors may adapt to proteostatic imbalance, potentially through enhanced proteasomal efficiency or other compensatory mechanisms. This dysregulated stress adaptation may not only underlie their aggressive phenotype but also reveal latent therapeutic vulnerabilities to agents targeting proteostasis. Collectively, these findings indicate that the risk signature captures fundamental biological characteristics of advanced myeloma, extending its potential utility from prognostication to the guidance of targeted therapeutic strategies.

To further characterize the tumor microenvironment, we applied the ESTIMATE algorithm, which revealed significant differences in immune scores between high- and low-risk groups. CIBERSORT analysis of 22 immune cell types showed that memory B cells and plasma cells were the most abundant, with memory B cells negatively correlated and plasma cells positively correlated with risk scores, consistent with MM progression (34). Furthermore, TIDE analysis indicated an increased potential for immune evasion in the high-risk group. However, as TIDE was developed using solid tumor datasets and MM exhibits a distinct immune microenvironment with differential responses to PD-1 blockade, these findings should be interpreted cautiously and considered exploratory. Preclinical studies in hematologic malignancies have demonstrated that combining pro-apoptotic agents, such as the cyclin-dependent kinase (CDK) inhibitor dinaciclib, with PD-1 blockade can synergistically enhance antitumor activity, overcome intrinsic resistance, and augment T-cell-mediated cytotoxicity through mechanisms including immunogenic cell death, T-cell proliferation, and dendritic cell activation (35). Accordingly, high-risk MM patients may benefit from multimodal immunotherapeutic strategies, pending further validation in MM-specific models and clinical contexts.

Our drug sensitivity analysis using the oncoPredict framework provides hypothesis-generating insights into potential differential therapeutic responses between risk groups. It should be noted, however, that these predictions are derived from large-scale pharmacogenomic databases, which may exhibit technical and platform-related variability and do not fully capture the spectrum of agents specifically developed for MM. Within this context, our results indicate that low-risk patients exhibited heightened sensitivity to compounds including WIKI4, WEHI-539, AZD6738, Linsitinib, AZD7762, and I-BRD9, whereas high-risk patients showed increased responsiveness to Doramapimod, SB216763, NU7441, SCH772984, JQ1, and IAP_5620. Several of these agents have mechanistic support in MM. Doramapimod enhances the anti-myeloma activity of conventional and novel drugs by inhibiting p38 MAPK and Hsp27 phosphorylation, overcoming resistance mechanisms, and disrupting stromal cell-derived cytokine support, thereby suppressing microenvironment-driven tumor growth (36). AZD7762, a Chk1/2 inhibitor, potentiates DNA-damaging agents such as bendamustine, melphalan, and doxorubicin in p53-mutated MM by abrogating checkpoint-mediated arrest and promoting apoptosis (37). Linsitinib, a dual IGF1R/INSR inhibitor, displays limited single-agent activity but synergistically enhances carfilzomib-induced cytotoxicity, suggesting utility in combination regimens (38). NU7441, a DNA-PK inhibitor, shows minimal single-agent efficacy but cooperates with MRE11 inhibition to block compensatory DNA repair, highlighting potential for dual-targeting strategies in MM with high replication stress (39). SB216763, a GSK-3 inhibitor, induces apoptosis and synergizes with bortezomib in an isoform-specific manner (40). JQ1, a BET inhibitor, suppresses MYC-driven transcription, attenuates MM growth, and modulates the tumor microenvironment (41).

These drug-risk associations suggest distinct pathway dependencies: low-risk tumors appear more vulnerable to WNT/chromatin regulation, anti-apoptotic (BCL-XL) inhibition, and checkpoint/IGF blockade (e.g., ATR/Chk1/2, Linsitinib), whereas high-risk tumors rely on MAPK-GSK3-MYC transcriptional programs and error-prone DNA repair mechanisms (e.g., Doramapimod, ERK/GSK3 inhibitors, JQ1, DNA-PK inhibition). These patterns are consistent with the immunosuppressive and microenvironmental features observed in high-risk patients and nominate rational, testable therapeutic combinations for each subgroup. Nonetheless, *in vitro* validation in MM cell lines and correlation with clinical outcomes (e.g., proteasome inhibitor or IMiD response) will be necessary in future studies to substantiate these findings.

Among the nine signature genes, three (ANXA2, HIF1A, and MAST4) have been previously implicated in MM (42–44), whereas the remaining six (MCPH1, TPST2, RANGAP1, KIF21B, ALG14, and PQLC3) remain largely uncharacterized in this context. Notably, several of these understudied genes have established mechanistic roles in other malignancies, providing insight into their potential oncogenic or tumor-suppressive functions and highlighting pathways for targeted investigation in MM. MCPH1, also known as BRIT1, serves as a multifaceted guardian of genomic integrity by promoting DNA damage sensing and repair, enforcing the G2/M checkpoint, limiting centrosome amplification, and stabilizing p53 through inhibition of MDM2-mediated ubiquitination; such functions plausibly link MCPH1 deficiency to genomic instability and cell cycle dysregulation in MM (45–47). TPST2 modulates the tumor immune microenvironment via tyrosine sulfation of interferon gamma receptor 1 (IFNGR1) at residue Y397, attenuating STAT1 signaling, reducing HLA expression and antigen presentation, and consequently diminishing tumor-infiltrating lymphocytes (48). In pancreatic ductal adenocarcinoma and breast cancer models, this mechanism alters sensitivity to anti-PD-1 therapy, indicating that receptor post-translational modification can reshape antitumor immunity (48, 49). RANGAP1 acts as a context-dependent regulator of chromosomal stability and signaling: its stabilization can promote MAPK pathway activation, whereas loss may precipitate chromothripsis, destabilize p53 and Rb, and accelerate tumorigenesis, thereby linking nucleocytoplasmic transport to genome integrity and oncogenic signaling (50–52). KIF21B, a kinesin motor protein, is upregulated in multiple cancers and enhances proliferation, migration, and survival via anti-apoptotic effects and PI3K/Akt pathway activation, while in certain contexts it also modulates Wnt/β-catenin signaling and antagonizes tumor-suppressive microRNAs. By contrast, PQLC3 and ALG14 are primarily supported by bioinformatic associations or non-oncologic literature: PQLC3 has been identified in prognostic and tumor mutational burden-related gene sets, whereas ALG14 is a canonical component of N-linked glycosylation (53–55). Their potential involvement in glucose metabolism and protein glycosylation in cancer remains hypothetical and warrants further experimental validation.

In summary, our study highlights disulfidptosis as a critical mechanism in MM and establishes a DRG-based prognostic model with robust predictive performance. The observed differences in the tumor immune microenvironment between risk groups provide mechanistic insights into MM progression and potential therapeutic vulnerabilities. Nevertheless, several limitations should be acknowledged. Key clinical variables, including International Staging System (ISS) and Revised ISS (R-ISS) stages, cytogenetic profiles, age, and treatment phase, were unavailable in the GEO cohorts, limiting formal adjustment for established prognostic factors. Despite this, the risk score consistently stratified patients across independent datasets, suggesting potential prognostic utility that warrants validation in clinically annotated cohorts. The size of our clinical validation cohort was also limited due to practical constraints, though we are actively pursuing future collaborations to expand this cohort. Finally, the functional roles of certain signature genes, particularly TPST2, MCPH1, ALG14, and PQLC3, remain incompletely understood in MM. Further mechanistic and clinical studies are required to elucidate their contributions to MM pathogenesis and drug resistance, which may inform the development of targeted therapeutic strategies.

## Data Availability

The datasets presented in this study can be found in online repositories. The names of the repository/repositories and accession number(s) can be found in the article/**Supplementary Material**.
