# Supplementary material for: Prognostic and therapeutic implications of disulfidptosis-related genes in multiple myeloma
Source: Front Immunol. 2025 Dec 1;16:1652179. doi: 10.3389/fimmu.2025.1652179 (PMC12702948; doi:10.3389/fimmu.2025.1652179)
Supplement: Supplementary file 1 [file DataSheet1.zip › Supplementary figures 1-10.DOCX]

Supplementary Information


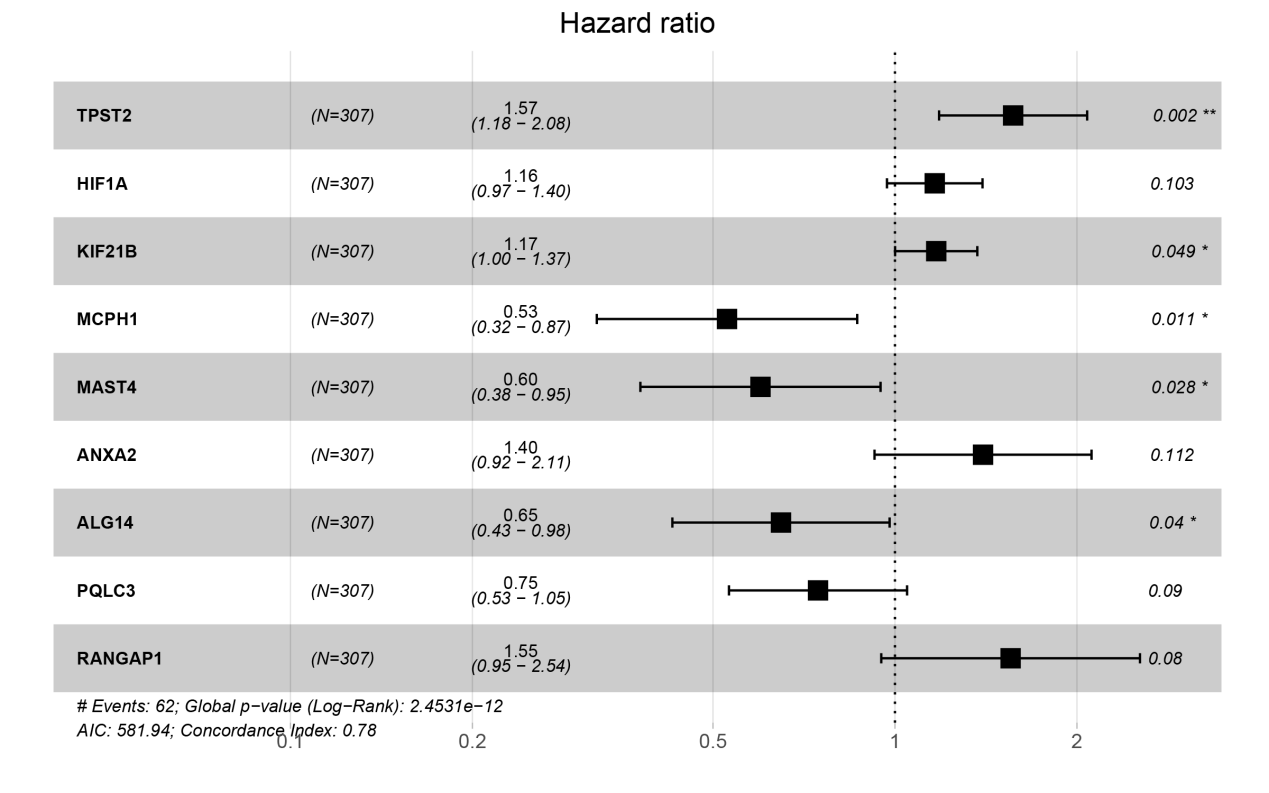


**Supplementary Figure 1** The forest plot of the multivariate regression analysis.


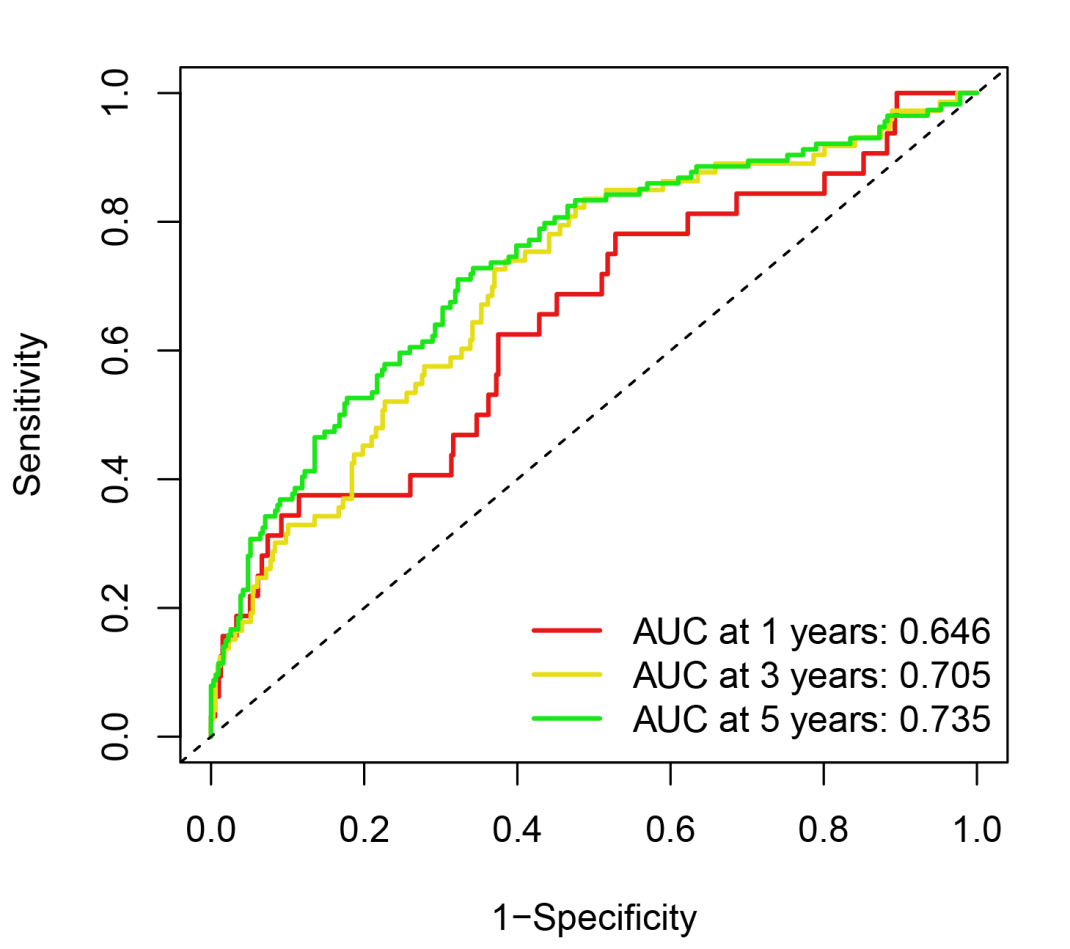


**Supplementary Figure 2** Validation of the prognostic model in the external cohort GSE136337 using ROC analysis.


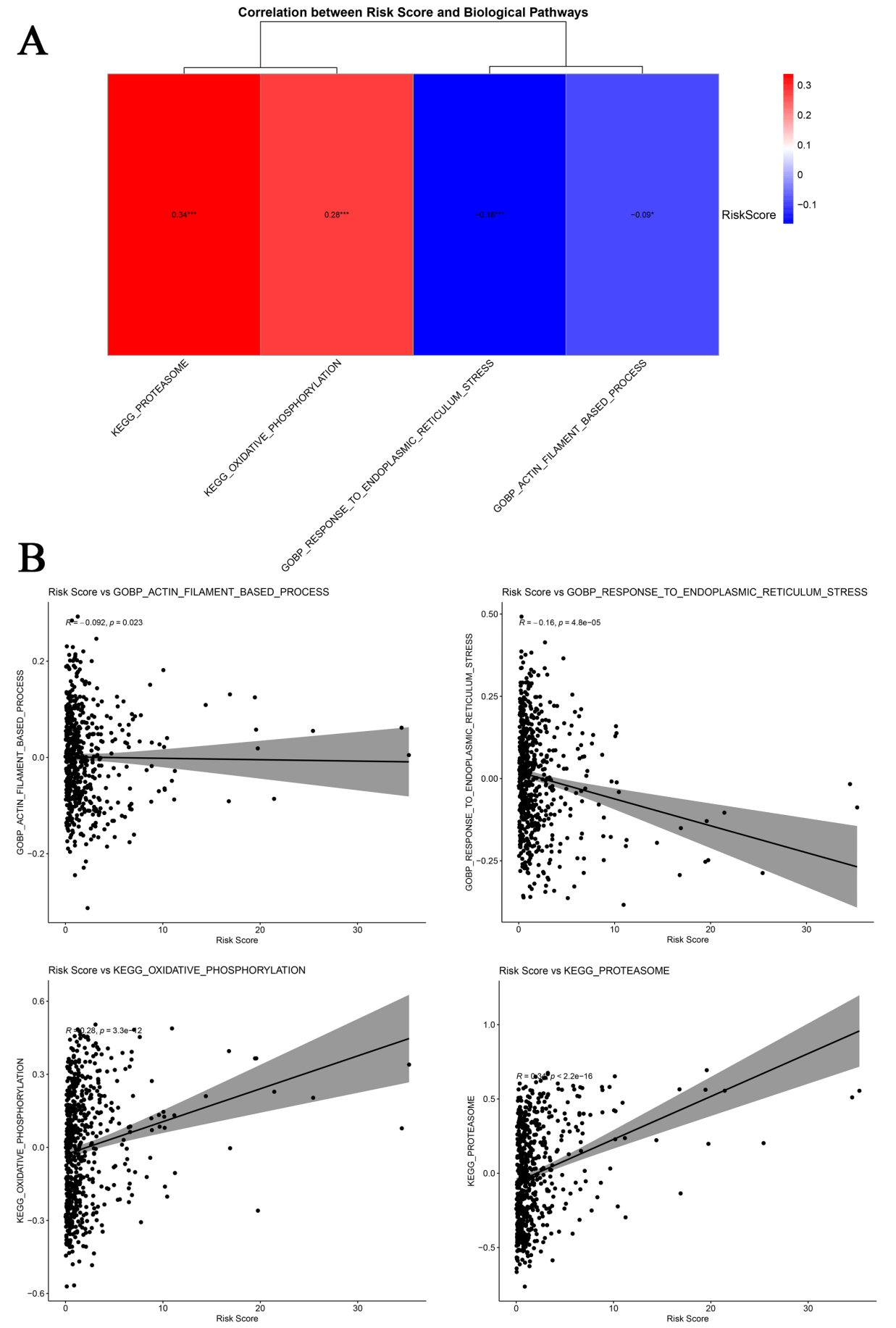


**Supplementary Figure 3** Correlation analysis between the disulfidptosis-related risk score and key pathway activities. **A** Heatmap of correlation coefficients for the risk score and key pathways. **B** Scatter plots of risk score versus pathway enrichment scores.

**
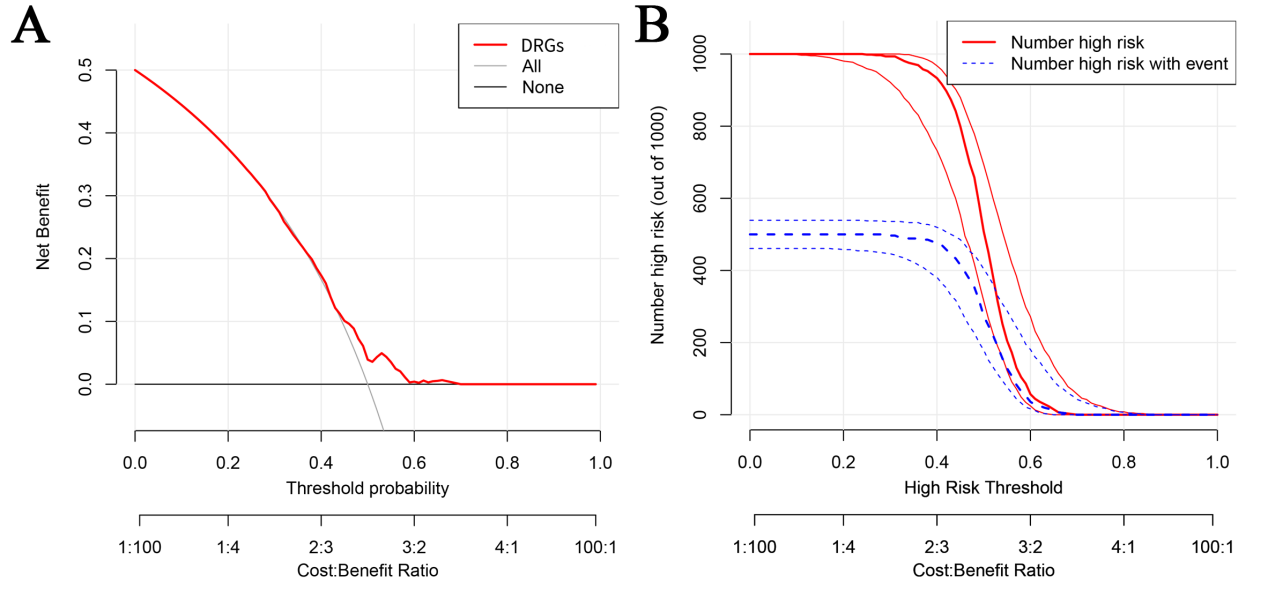
**

**Supplementary Figure 4** Evaluation of the clinical utility of the prognostic nomogram. **A** Decision curve analysis of the nomogram. **B** Clinical impact curves of the nomogram.


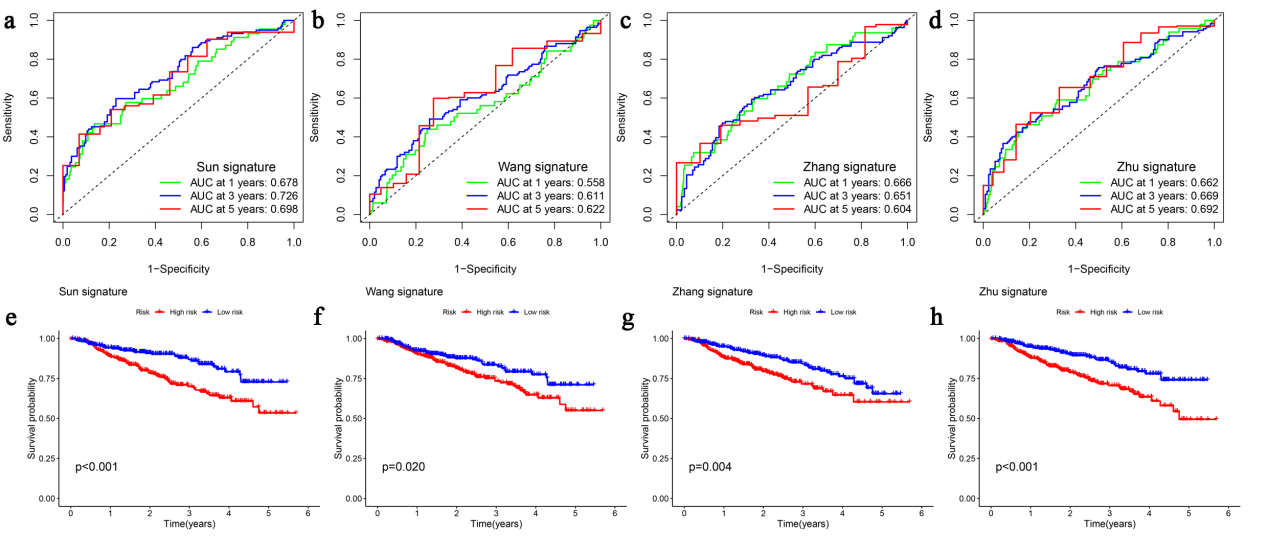


**Supplementary Figure 5** Comparative prognostic capabilities of the disulfidptosis model with four other models. **A-D** Survival rates in four MM models. **E-H** ROC analysis in four MM models.


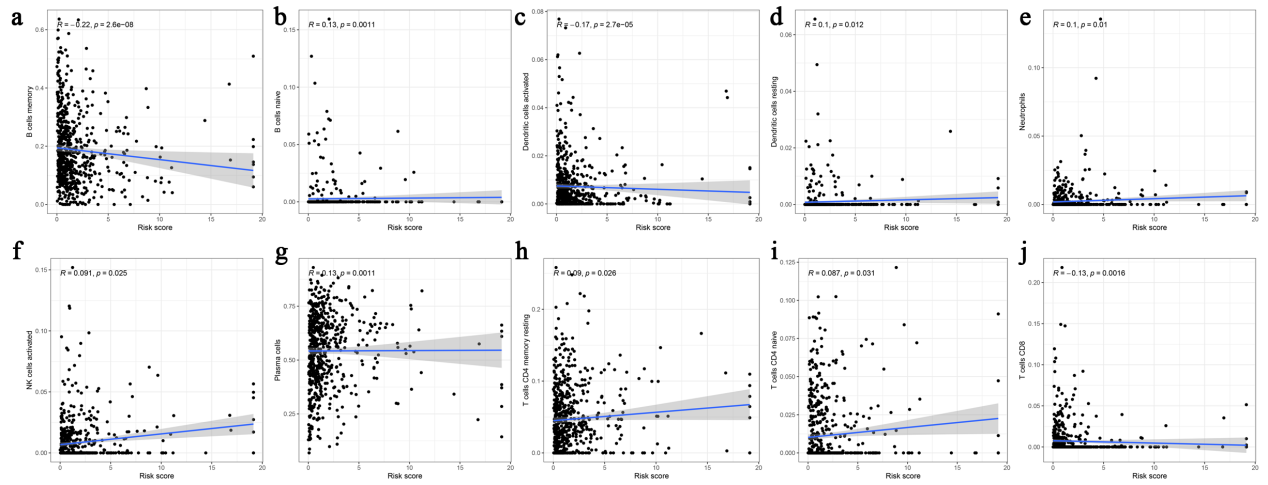


**Supplementary Figure 6** Correlation analyses between contents of immune cells and risk scores.


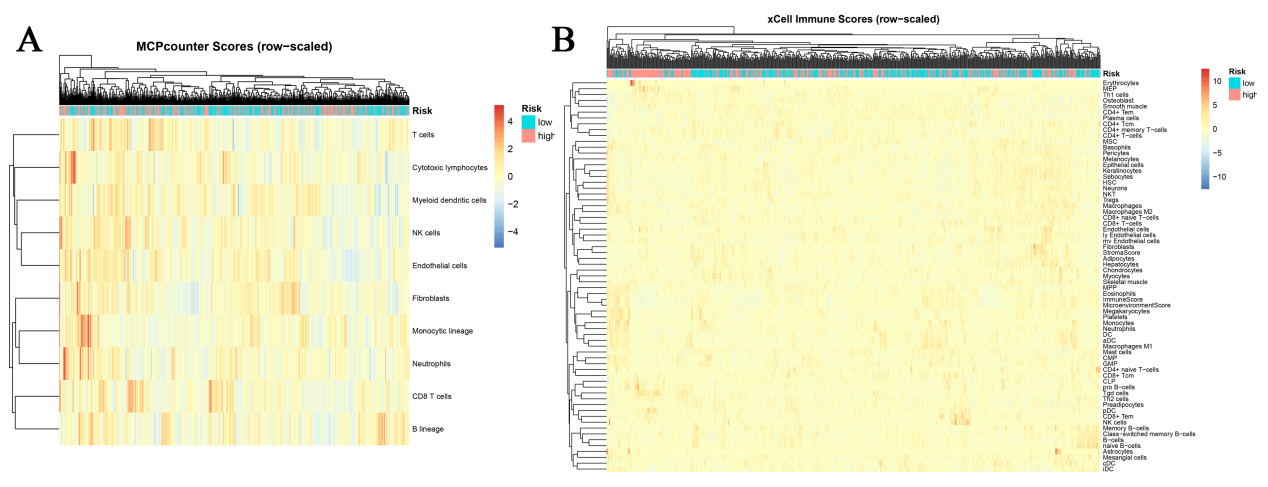


**Supplementary Figure 7** Characterization of the tumor immune microenvironment in samples. Immune cell infiltration profiles were estimated using (**A**) xCell and (**B**) MCP-counter algorithms.


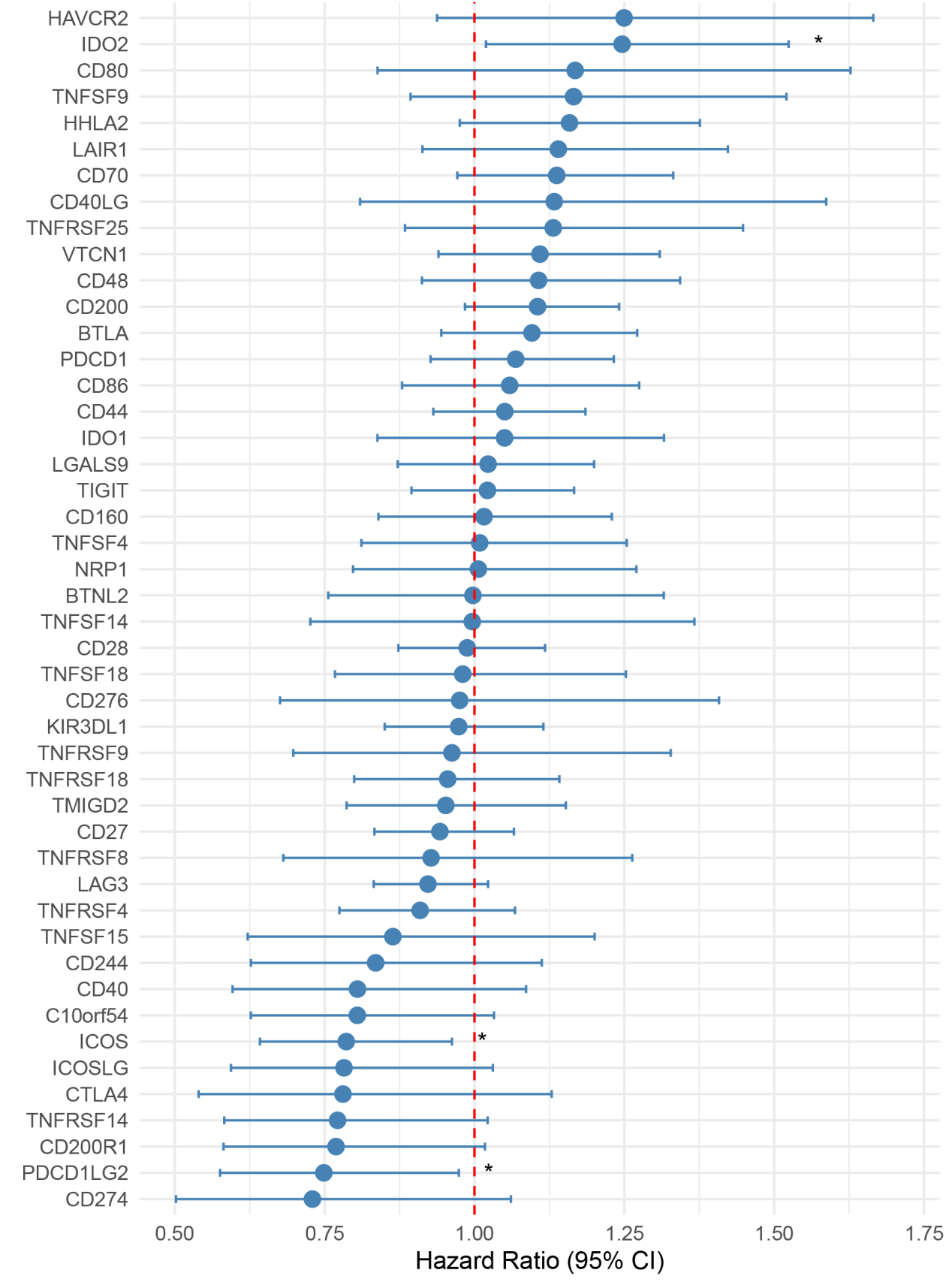


**Supplementary Figure 8** Forest plot of multivariable Cox regression analysis for immune checkpoint genes.

**
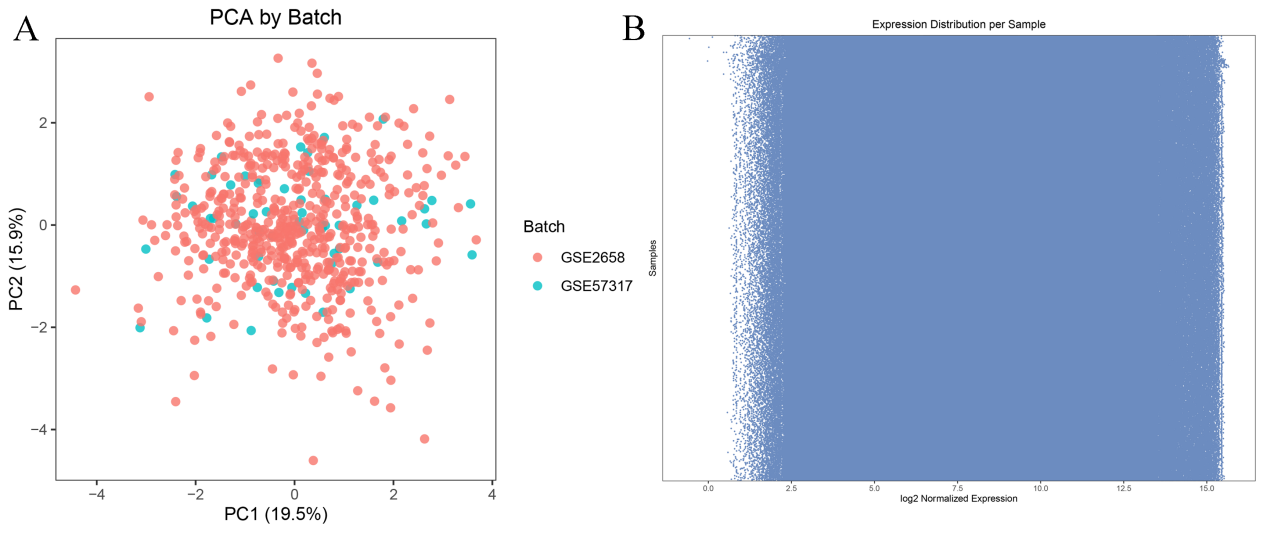
**

**Supplementary Figure 9** Quality control, batch effect assessment, and evaluation of data consistency. **A** PCA plot for batch effect assessment. **B** Boxplot of normalized gene expression distribution across all samples.


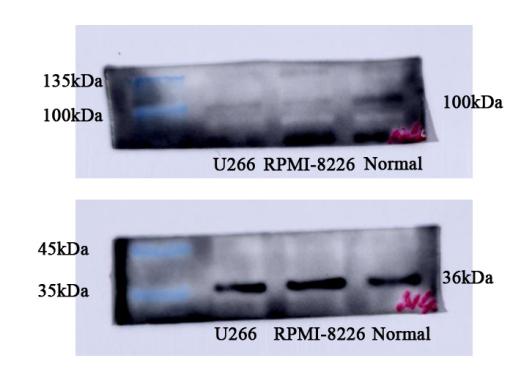


**Supplementary Figure 10** Original blots of Figure 9C.
