# Supplementary material for: Prognostic and therapeutic implications of disulfidptosis-related genes in multiple myeloma
Source: Front Immunol. 2025 Dec 1;16:1652179. doi: 10.3389/fimmu.2025.1652179 (PMC12702948; doi:10.3389/fimmu.2025.1652179)
Supplement: Supplementary file 1 [file DataSheet1.zip › Additional file 2.DOCX]

| DRGs | Official full name | Primary function | Literature on the correlation between genes and disulfidptosis |
| --- | --- | --- | --- |
| ACTB | actin beta | actin filament formation and cytoskeletal structure maintenance | doi:10.1038/s41598-024-59243-9. |
| ACTN4 | alpha-actinin-4 | actin filament crosslinking and cytoskeletal organization | doi: 10.3233/CBM-230276. |
| CAPZB | capping actin protein of muscle Z-line subunit beta | actin filament capping and regulation of filament dynamics | doi: 10.1186/s10020-024-01024-1. |
| CD2AP | CD2 associated Protein | actin cytoskeleton organization and membrane trafficking | doi: 10.1007/s12672-025-02308-6. |
| DSTN | destrin | actin filament depolymerization and severing | doi: 10.3389/fimmu.2024.1288240. |
| FLNA | filamin A | actin filament crosslinking and cytoskeletal organization | doi: 10.1186/s11658-025-00761-3. |
| FLNB | filamin B | actin filament crosslinking and cytoskeletal organization | doi: 10.1016/j.intimp.2024.111605. |
| GYS1 | glycogen synthase 1 | rate-limiting enzyme in glycogen synthesis | doi: 10.1002/mco2.502. |
| INF2 | inverted formin 2 | actin filament nucleation and remodeling | doi: 10.1002/advs.202408556. |
| IQGAP1 | IQ motif containing GTPase activating protein 1 | actin cytoskeleton regulation and cell–cell adhesion | doi: 10.18632/aging.205688. |
| LRPPRC | leucine rich pentatricopeptide repeat containing | mitochondrial mRNA stability and translation regulation | doi: 10.3389/fimmu.2024.1454730. |
| MYH10 | myosin heavy Chain 10 | actomyosin contractility and cytoskeletal organization | doi: 10.1038/s41598-025-89128-4. |
| MYH9 | myosin heavy Chain 9 | actomyosin contractility and cell migration | doi: 10.1016/j.tranon.2024.102091. |
| MYL6 | myosin light Chain 6 | regulation of myosin ATPase activity and actin–myosin interaction | doi: 10.1515/med-2024-0929. |
| NCKAP1 | NCK associated protein 1 | actin filament branching and lamellipodia formation | doi: 10.1007/s12672-024-01612-x. |
| NDUFA11 | NADH:ubiquinone oxidoreductase subunit A11 | mitochondrial complex I assembly and electron transport | doi: 10.3389/fnins.2024.1505493. |
| NDUFS1 | NADH:ubiquinone oxidoreductase core subunit S1 | mitochondrial complex I catalytic activity and electron transfer | doi: 10.1016/j.nbd.2025.106789. |
| NUBPL | nucleotide binding protein-like | mitochondrial complex I assembly and iron–sulfur cluster incorporation | doi: 10.1016/j.heliyon.2024.e37638. |
| PDLIM1 | PDZ and LIM domain 1 | actin cytoskeleton organization and cell adhesion regulation | doi: 10.3390/biomedicines12081840. |
| RPN1 | ribophorin I | N-linked glycosylation and protein processing in the endoplasmic reticulum | doi: 10.1016/j.heliyon.2024.e31875. |
| SLC3A2 | solute carrier family 3 member 2 | amino acid transport and integrin-mediated cell adhesion | doi: 10.1007/s12672-024-01612-x. |
| SLC7A11 | solute carrier family 7 member 11 | cystine/glutamate antiporter activity and redox homeostasis | doi: 10.1007/s12672-024-01612-x. |
| TLN1 | talin 1 | integrin activation and actin cytoskeleton linkage | doi: 10.2147/JIR.S525114. |
| OXSM | 3-oxoacyl-ACP synthase, mitochondrial | mitochondrial fatty acid elongation and lipid metabolism | doi: 10.1038/s41598-025-93656-4. |

**Additional File 2** Curated list of 24 disulfidptosis-related genes (DRGs) with literature support.
